# Adolescent idiopathic scoliosis (AIS): a multifactorial cascade concept for pathogenesis and embryonic origin

**DOI:** 10.1186/s13013-016-0063-1

**Published:** 2016-01-30

**Authors:** R. Geoffrey Burwell, Emma M. Clark, Peter H. Dangerfield, Alan Moulton

**Affiliations:** Centre for Spinal Studies and Surgery, Queen’s Medical Centre, Nottingham University Hospitals Trust, Nottingham, UK; Academic Rheumatology, Musculoskeletal Research Unit, University of Bristol, Bristol, UK; University of Liverpool & Staffordshire University, Staffordshire, UK; Department of Orthopaedic Surgery, King’s Mill Hospital, Mansfield, UK

**Keywords:** Scoliosis, Aetiology, Pathogenesis, Fat, Muscle, Embryology, Epidemiology

## Abstract

This paper formulates a novel multifactorial Cascade Concept for the pathogenesis of adolescent idiopathic scoliosis (AIS). This Concept stems from the longitudinal findings of Clark et al. (J Bone Miner Res 29(8):1729-36, 2014) who identified leptin body composition factors at 10 years of age associated with a scoliosis deformity found at 15 years. We interpret these findings in the light of some concepts for AIS pathogenesis. In particular, we speculate that the leptin body composition effect is linked to central nervous system development and the initiation of the asynchronous neuro-osseous growth mechanism that involves the creation of a neuraxis tether of relative anterior vertebral overgrowth. The latter mechanism in combination with age and gender-related anatomical variants of vertebral backward tilt (dorsal shear concept), human upright posture, adolescent growth factors, Hueter-Volkmann effect in vertebrae and vertebral bone mass abnormalities, lead to AIS, possibly both initiation and progression of scoliosis curvatures. Being multifactorial, while the Cascade Concept cannot be tested for all its components, some components should be testable by the method of numerical simulation.

Clark et al. (J Bone Miner Res 29(8):1729-36, 2014) also suggested the origin of scoliosis was in the embryonic stages of life from cell types, including adipocytes and osteoblasts, derived from the same progenitor cells, and myoblasts from mesodermal somites. The involvement of cell types from different developmental origins suggests a process acting in embryonic life at a similar time, probably environmental, as previously proposed from anthropometric studies. As a Complex disease, AIS will involve genetic, environmental and life style factors operating in development and growth; this possibility needs evaluating in epidemiological studies.

## Background

**Fig. 1:**
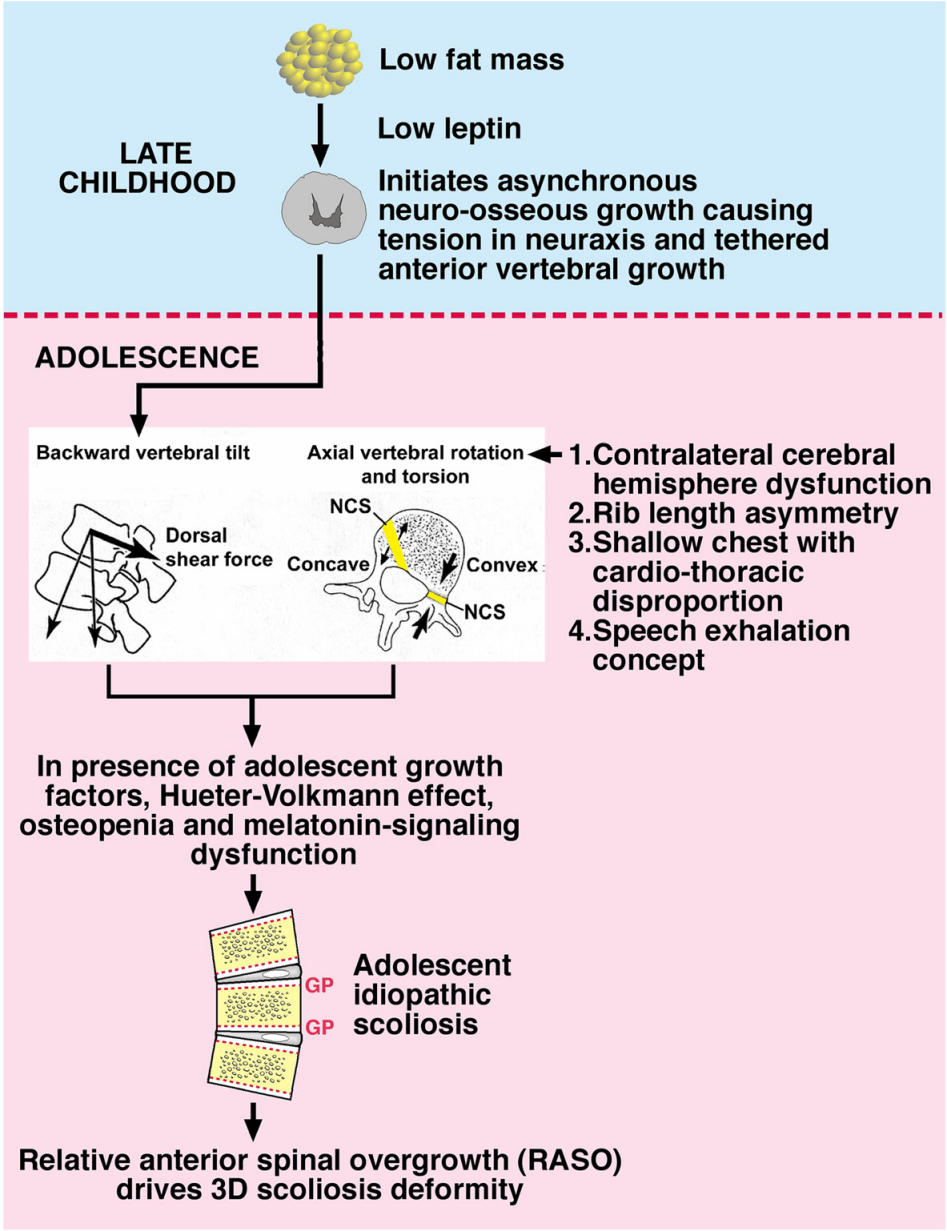
Cascade Concept for AIS pathogenesis based on the findings of Clark et al. [4] that place adipose tissue and energy control in relation to the predisposition to AIS. It is speculated that leptin is linked to human central nervous system development, asynchronous neuro-osseous growth mechanism, and the dorsal shear mechanism. NCS = neurocentralsynchondrosis, GP = growth plate

While there is no agreed theory for the pathogenesis of adolescent idiopathic scoliosis (AIS), several concepts attempt to do so, focusing on specific pathogenetic processes [1–3]. Here, we suggest a novel speculative multifactorial Cascade Concept for AIS pathogenesis. It stems from the longitudinal findings of Clark et al. [4] which we interpret here in the light of some pathogenetic concepts for AIS (Fig. 1). Preliminary accounts of the concept have been presented [3, 5, 6].

### The Avon longitudinal study of parents and children (ALSPAC) and idiopathic scoliosis

Clark et al. [4] identified factors at 10 years of age that were associated with a scoliosis deformity identified at 15 years of age. These factors are low fat mass, low lean mass, low circulating leptin and high circulating adiponectin levels. We speculate that this leptin body composition effect in AIS is linked to central nervous system development, as shown in mice for the brain [7] and, in particular to impaired neuraxis growth as it stretches in adapting to vertebral column growth as the child grows in height.

## Components of cascade concept

### Leptin and central nervous system (CNS) development

We speculate that the leptin body composition effect of Clark et al. [4] links spinal cord development to the asynchronous neuro-osseous growth concept for AIS pathogenesis [8] for four reasons. Firstly, the decreased brain size of the *ob/ob* mouse is evidently due to a developmental defect that can be corrected by leptin administration, indicating that leptin plays a role in brain growth and development [7]. Secondly, leptin in *ob/ob* mice is reported to regulate the myelination of oligodendrocytes [9]. Thirdly, in primates, brain mass increases linearly with spinal cord mass, while neuron number in the brain increases with neuron number in the spinal cord raised to the power of 1.7 [10]. Fourthly, the low leptin effect on the CNS in AIS subjects [3, 11] may explain the different thinning pattern of the cerebral cortex observed in patients with AIS during adolescence, which may be primary (i.e. pathogenetic) or secondary (i.e. adaptation) to the development of scoliosis [12].

### Asynchronous neuro-osseous growth mechanism for AIS pathogenesis

Using the multi-planar reconstruction technique of magnetic resonance imaging, Chu et al. [8]:

“....investigated the relative length of spinal cord to vertebral column, including ratios, in 28 girls with AIS (mainly thoracic or double major curves) and 14 age-matched normal girls. Also evaluated were cerebellar tonsillar position, somatosensory evoked potentials (SSEPs), and clinical neurological examination. In severe AIS compared with normal controls, the vertebral column is significantly longer without detectable spinal cord lengthening. They speculate that anterior spinal column overgrowth relative to a normal length spinal cord exerts a stretchintethering force between the two ends cranially and caudally, leading to the initiation and progression of thoracic AIS. They support and develop the Roth-Porter concept of uncoupled neuro-osseous growth in the pathogenesis of AIS which now they prefer to term ’asynchronous neuro-osseous growth’.

Lengths of the vertebral column were measured from the tip of C2 down to the inferior end plate of L5, and the spinal cord from the tip of C2 down to the conus medullaris [8].

### Conus medullaris and asynchronous neuro-osseous growth – hypothesis of impaired growth response of neuraxis to stretch with spinal growth creates a tether to anterior spinal overgrowth

In AIS subjects, the mean and distribution of conus medullaris locations are similar to controls [13, 14]. During normal development, because of different relative rates of growth of the vertebral column and spinal cord, the level of termination of the spinal cord is constantly changing, particularly prenatally [15]. In the normal spine of children, the conus is reported to reach its adult level by 2 years of age at an average position of L1 to L2 [15, 16]. We interpret subsequent spinal cord and cauda equina lengthening as a neural stretch adaptation to the linear growth of vertebrae. In the scoliosis subjects, measured by Chu et al. [8], where there was anterior spine overgrowth, the scoliosis was attributed to tethering of the relative anterior vertebral overgrowth by a normal length spinal cord (caudal part of neuraxis); also the seat of pathology which may extend into the brain stem neuraxis [17]. We explain the normal length spinal cord as resulting from impaired neuraxis growth in response to stretch due to a low leptin effect on neuraxis growth from age 10 years or earlier. This accounts for the finding of a spinal cord of normal length in AIS subjects with anterior vertebral [8] and skeletal overgrowth [18] driven by the hormones of puberty.

### Cranial expression of tension in the neuraxis

Though not reported in the AIS subjects studied by Chu et al. [8], in the light of other findings [13, 14], the conus location was probably normal. If this be the case, then their finding of a normal length spinal cord with anterior spinal overgrowth suggests that the tension created in a relatively short spinal cord (neuraxis tether) by anterior vertebral growth is expressed, not caudally at conus level, but cranially in the upper cervical cord and medulla oblongata (as disturbed white matter) [17] and at the craniocervical junction (as low-lying cerebellar tonsils) [19].

The evidence for thoracic AIS [8] is consistent with the view that after 2 years of age, the cauda equina stretches with lumbar spine growth to produce a normal conus termination level. In contrast, the spinal cord neuraxis does not stretch fully and grow with the cervicothoracic spine, which causes traction on the upper cervical cord and brain stem. In the latter connection Kong et al. [17] write:

“The findings from this study are in agreement with previous findings showing abnormal somatosensory evoked potential readings occurring only above the C5-6 level in patients with adolescent idiopathic scoliosis; these findings might partially explain the pathophysiology of the neural pathway involved”.

### Cascade concept and normal length of spinal neuraxis

This interpretation applies to the Cascade Concept in which low circulating leptin levels impair growth of the spinal neuraxis. But how does this explain the reported normal length of the spinal cord [8]? Should the initial growth potential of the spinal neuraxis be coupled to that of the anterior spine for overgrowth [8], then impaired spinal neuraxis growth could produce a normal length spinal cord with anterior spinal overgrowth as reported by Chu et al. [8].

### Dorsal shear concept for AIS pathogenesis

The speculation of Chu et al. [8] that the initiation and progression of AIS results from anterior vertebral column overgrowth through a lordoscoliotic maladaptation of the spine to the subclinical tether of a relatively short spinal cord, suggests that the neuraxis tethering may also act by altering the backward tilt of vertebrae in the lower spine within the dorsal shear concept of pathogenesis for AIS [20].

Castelein et al. [20] postulated that:

“… dorsal shear forces, acting exclusively upon specific regions of the human spine, contribute to rotational instability of the spine. Asymmetric loading of the posterior parts of the vertebrae then would lead to asymmetrical growth in all three planes of specific parts of vertebrae, according to the Hueter-Volkmann’s law. Asymmetrical growth of the neurocentral cartilage of the vertebra, for instance, has been shown to lead to AIS-like deformities in growing pigs, and could explain the development and progression of the deformity in humans”.

Schlosser et al. [21] conclude:

“.....the spines of girls during the growth spurt are more posteriorly inclined, and thus rotationally less stable, compared to boys at the same stage of development, as well as compared to girls after the growth spurt. This may explain why initiation and progression of adolescent idiopathic scoliosis are more prevalent in girls around puberty”.

We speculate that the leptin body composition effect is linked to central nervous system development and the asynchronous neuro-osseous growth mechanism. The latter, in combination with human upright posture, age and gender-related anatomical variants of vertebral backward tilt (dorsal shear concept), adolescent growth factors, the Hueter-Volkmann effect in vertebrae and vertebral bone mass abnormality, lead to AIS, possibly both initiation and progression of scoliosis curvatures.

### Hueter-Volkmann law

The Hueter-Volkmann Law states that increased mechanical compression acting on growth plates impairs skeletal growth and reduced loading increases skeletal growth. Several concepts of AIS pathogenesis use the Hueter-Volkmann effect in their mechanisms [1, 22–24], each of which requires an initiating mechanism to deform the spine. The Cascade Concept for AIS includes the Hueter-Volkmann effect in its pathomechanisms (Fig. 1). This mechanism lies within the field of mechanobiology that in the skeleton includes the effects of Hueter-Volkmann, Pauwels and Wolff [24].

### Vertebral bone mass

Once a scoliogenic mechanism, such as in the Cascade Concept, has initiated a spinal deformity, the presence in that subject of any reduced vertebral bone mass from known, (e.g. osteopenia, [25], melatonin-signalling dysfunction [26, 27], vitamin D [28] and possible calcium [29] deficiency), or from unknown causes, will facilitate progression of the scoliosis deformity.

In healthy subjects, both low fat mass and low lean mass are independent predictors of low bone mass [30, 31]. In individuals with scoliosis, Clark et al. [4] linked low lean mass to abnormalities of paravertebral muscle histology and electromyographic function. Here, we interpret the associations between low fat mass, low lean mass, bone mass and scoliosis as causal and determined in embryonic life.

### Developmental axial vertebral rotation

The normal human spine in the transverse plane is not symmetrical. Between infancy and adolescence in the mid and lower thoracic spine, axial vertebral rotations (AVRs) convert from left to right [32, 33]. We term this the left-right AVR conversion [34]. It reflects the most prevalent curve patterns in idiopathic scoliosis at different ages [32, 33].

### Hypothesis of oscillating axial torsion and AVR conversion in normal development

We formulated the oscillating axial torsion hypothesis [35] in a study of upper limb length asymmetry in idiopathic scoliosis [36, 37]. The hypothesis is based on torsions of neural and skeletal structures in normal, pre-and post-natal human development. The postnatal left-right AVR conversion of the normal spine is contained within this oscillating axial torsion hypothesis where it is viewed as being determined by intrinsic developmental processes within growing axial structures; this is in contrast to the AVR conversion being interpreted as a mechanical adaptation of the spine to visceral asymmetry in the trunk [32, 33].

This controversy of mechanisms determining the AVR conversion was identified by Schlosser et al. [38] who suggested that while neurocentral synchondrosis (NCS) asymmetry and their fusion is related to pre-existent rotation of the spine; whether the NCS asymmetry is the cause, or is caused by, the pre-existent vertebral rotation, could not be determined. This controversy needs further consideration because, if normal intrinsic mechanisms determine the AVR conversion, then abnormality of these intrinsic mechanisms could be scoliogenic, not only in adolescence, but also in infancy [34].

### Putative thoracic axial rotation factors

The Cascade Concept for AIS pathogenesis posits that the quantum of developmental axial vertebral rotation can be increased by one of at least four factors (Fig. 1): 1) contralateral cerebral hemisphere dysfunction [39, 40]; 2) rib length asymmetry [41, 42]; 3) shallow chest acting through the ribs [43]; and 4) putative speech exhalation-associated scoliogenic mechanism acting through the ribcage [44]. We suggest that any such increase of axial vertebral rotation (transverse plane) when combined with backward vertebral tilt (sagittal plane) within the dorsal shear concept for AIS pathogenesis [20, 21] will facilitate the development of a scoliosis deformity.

## Embryonic origin of cascade concept for AIS pathogenesis

**Fig. 2:**
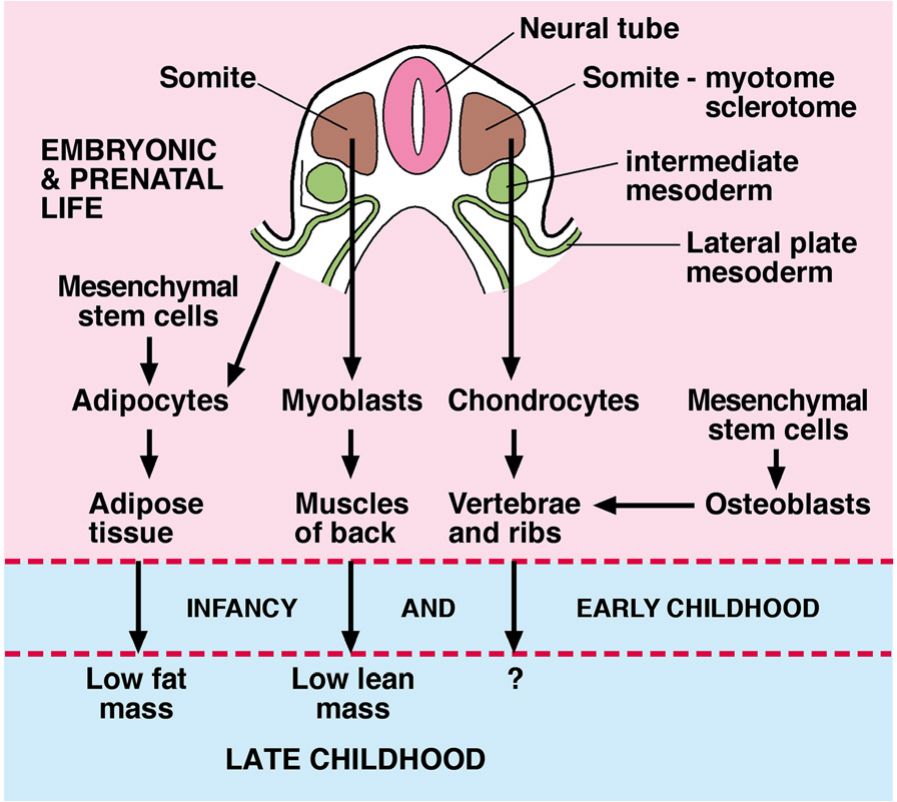
Diagram from the description of Clark et al. [4] to show embryonic origin of cell types from progenitor cells suggested to be involved in AIS development. The cell lineages are from Gilbert [53] and Chau et al. [45]

To explain the association of components of body composition identifiable before the onset of scoliosis, Clark et al. [4] suggest that the origin of scoliosis affects a cluster of cell types, namely adipocytes and osteoblasts derived from the same progenitor cells (mesenchymal stem cells), and myoblasts derived from different progenitor cells (somitic myotome) (Fig. 2). Recent research suggests consideration is also given to visceral white adipose tissue arising from lateral plate mesoderm [45]. The involvement of cell types from different developmental origins suggests a process acting in embryonic life, probably environmental. This origin was also suggested from anthropometric studies [36].

We focus here on low fat mass and low leptin in relation to the Cascade Concept of AIS pathogenesis. How may abnormalities of adipocytes and other cell types arise? Although long form leptin receptor mRNA was expressed in the brain, pituitary, and other tissues, it was not detected in the spinal cord of pigs (gilts) [46]. This finding suggests the possibility that any such leptin effect on spinal cord growth may be indirect involving unknown factors.

## Sporadic AIS as a complex disease – genes and environment

Sporadic AIS has been termed a Complex disease by not following the classical mode of Mendelian inheritance and for other reasons [3]. Complex diseases are caused by a combination of genetic, environmental, and lifestyle factors, mostly not yet identified; the vast majority of diseases fall into this category [47].

As a Complex disease, sporadic AIS will involve genetic, environmental and life style factors in development and growth [3]. Although environmental factors are involved in AIS aetiopathogenesis, no specific factor(s) has been identified [48, 49]. A possible environmental mechanism has been evaluated in the first year of life; it involves a time lag (time-dependent reaction) between exposure and expression of the scoliosis phenotype [50].

## Conclusions

Here we focus on two basic questions posed by the research of Clark et al. [4]:

- Does low fat mass and low leptin levels present at 10 years impair neuraxis growth between 10 and 15 years of age?
- May environmental factors acting on a susceptible genotype in early embryonic life determine AIS years later?

1. The observations of Clark et al. [4] relating to the scoliosis phenotype and endophenotype need confirming in other population or disease cohorts.
2. Rather than study the phenotype and endophenotype from birth, or even conception, knowledge may be facilitated by evaluating familial AIS in younger unaffected siblings of AIS girls in coordinated multicentre longitudinal studies.
3. Some biomechanical aspects of the Cascade Concept may be testable by *numerical simulation*. This method that involves three-dimensional reconstructions of the scoliotic spine has been used to study the effects of gravity loads, disc mechanical stiffness and anterior vertebral growth [51].
4. The *numerical simulation* method may be applied to components of the scoliotic spine in the sagittal and transverse planes, relating to both the *asynchronous neuro-osseous growth mechanism* and *the dorsal shear concept for AIS pathogenesis.*
5. As in the research of McMaster et al. [50] which focused on the first year of postnatal life, the process leading, after a time lag, to AIS might also affect susceptible embryos. This needs evaluating in epidemiological studies. Further epidemiological research is needed, including early pregnancy. The latter should include consideration of the perinatal microbiome [52].
